# Focus group interviews reveal reasons for differences in the perception of disease activity in rheumatoid arthritis

**DOI:** 10.1007/s11136-016-1369-4

**Published:** 2016-07-21

**Authors:** Margot J. M. Walter, Adriaan van’t Spijker, Annelieke Pasma, Johanna M. W. Hazes, Jolanda J. Luime

**Affiliations:** 1 Department of Rheumatology, Erasmus Medical Center, University Medical Center Rotterdam, Postal Box 2040, 3000 CA Rotterdam, The Netherlands; 2 Department of Psychiatry, Section Medical Psychology and Psychotherapy, Erasmus Medical Center, University Medical Center Rotterdam, Rotterdam, The Netherlands

**Keywords:** Qualitative research, Rheumatoid arthritis, Discordance, Disease activity

## Abstract

**Objective:**

Doctors frequently see patients who have difficulties coping with their disease and rate their disease activity high, despite the fact that according to the doctors, the disease activity is low. This study explored the patients’ perspectives on this discordance that may help to understand why for some patients, usual care seems to be insufficient.

**Methods:**

In our qualitative study we conducted focus group interviews where questions were used as a guideline. Transcripts were analyzed using inductive thematic analysis.

**Findings:**

Twenty-nine patients participated in four focus groups. Participants could not put their finger exactly on why doctors estimated that their disease activity was low, while they experienced high levels of disease activity. During the in-depth focus interviews, seven themes emerged that appeared related to high experienced disease activity: (1) perceived stress, (2) balancing activities and rest, (3) medication intake, (4) social stress, (5) relationship with professionals, (6) comorbidity, and (7) physical fitness.

**Conclusion:**

When patients were asked why their view of their disease activity was different from that of their physician, seven themes emerged. The way participants coped with these themes seemed to be the predominant concept. Specific interventions that focus on one or more of the reported themes and on coping may improve not only the quality of life of these patients but also the satisfaction with the patient–doctor relationship for both parties.

## Introduction

In rheumatoid arthritis (RA), patients and physicians do not always rate disease activity equally [1–4]. Despite the fact that treatment is regarded effective on commonly used disease activity measures (e.g., disease activity score, clinical disease activity index), about one-third of patients with low disease activity report high levels of pain, functional disability and fatigue [1–7]. This difference is undesirable, as it may affect the patient’s satisfaction, adherence to treatment [8, 9], and outcome [8]. Differences between patients and physicians regarding the perception of disease activity are not well understood and may relate to various factors.

In the context of shared decision making and patient-centered care, it is important to know the patients’ thoughts about the high disease activity that they perceive and this possible discordance. Data from cohort studies suggest that in the perception of the patient, the most relevant disease activity parameters are pain and fatigue [6], while for the physician, the most important parameter is the number of swollen joints [4, 10]. Moreover, previous studies suggest a role for factors influencing discordance such as education, health literacy, and the concurrent presence of depression [2, 4, 11]. Furthermore, qualitative studies showed that pain, mobility, fatigue, physical capacity, and well-being are seen as an important outcome for patients [12, 13].

A better understanding of factors—according to the individual patient—that influence the high self-reported disease activity may help to understand why for some patients, usual care seems to be insufficient. Therefore, the aim of this study was to explore the patient’s perspective on the patient–physician discordance with regard to disease activity in rheumatoid arthritis.

## Methods

To explore patients’ perspectives in breadth and depth on the discordance of the disease activity between patients and physicians, a qualitative study was performed by using focus group interviews. Focus groups allowed an interactive discussion on the topic of discordance. This method enabled researchers to explore the experiences, concerns, collective understanding, and opinions of participants by discussing specific topics related to discordance of disease activity and generate data [14].

### Initial cohort

Patients from the RAPPORT study (Rheumatoid Arthritis Patients rePort Onset ReacTivation), an observational cohort [15], were invited to participate in this study by letter. In brief, RA patients were eligible for this study if they were aged 18 years or older and were able to read and write Dutch. Further study details can be found in Walter [15]. Of the initial 159 RAPPORT study patients, 82 patients (52 %) were willing to participate. No significant differences between responders and non-responders were found with regard to demographic characteristics, previous disease activity, and previous patient-reported outcome (PRO) scores [15]. The disease activity was measured with the disease activity score (DAS28). This score ranges from 0 to 10 containing swollen joints, tender joints, visual analog scale (VAS) global, and erythrocyte sedimentation rate (ESR), where a higher score indicates a higher disease activity.

Patients were asked to complete web-based questionnaires (Health Assessment Questionnaire/HAQ, Rheumatoid Arthritis Disease Activity Index/RADAI and Visual analog scale/VAS fatigue) three times, at 3-week intervals, and were clinically assessed by their consultant rheumatology once. Based on the online PROs and disease activity as rated by the physicians, 29 patients were identified as being discordant. According to regulations in the Netherlands (WMO), approval from the ethical review board was not needed. All patients gave written informed consent before inclusion in the focus groups.

### Patient selection for the focus group interviews

Patients who had a discrepancy between patient-reported outcomes (PROs) and physician-assessed disease activity were invited to take part in focus groups. Patients were regarded as discordant if they had low disease activity according to the physician and a high PRO score (HAQ > 1 [16], RADAI > 2.2 [17] and VAS fatigue > 50) for two or three consecutive time points. The interview schedule was devised after discussions between a clinical psychologist, nurse practitioner, and an epidemiologist as well as a literature search (Table 1). The duration of the sessions was 1–1.5 h. They were led by a male psychologist. The moderator introduced himself as being interested in this topic and emphasized the confidentiality of the interviews. All interviews were audio taped, transcribed verbatim, and anonymized.

**Table 1:** Interview questions

*General question*
What makes you say that your disease activity is high, while the rheumatologist indicates that the disease activity is low?
*Additional questions*
What do you do when your disease activity is high?
Was your intervention effective?
Do you believe there are influencing factors that can reduce your disease activity?

### Data analysis

Data analysis was based on grounded theory [18]. We adopted not a constructivist approach to grounded theory [19], but adopted a more thematic analysis of the data. After each interview, emergent themes were identified. The principle of data saturation was used. Interviews were held until themes and categories in the data become repetitive and redundant, such that no new information could be gleaned [20, 21]. At this point, saturation has been reached, and depth and breadth of the information were achieved [21]. No new concepts emerged after four focus groups (data saturation). Emergent themes from the first interviews were incorporated into the next interviews. When themes were not discussed spontaneously, groups were asked explicitly about themes from previous interviews. After all interviews were held, the transcripts were read and re-read by the principal investigator to gain an overall understanding of the interviews. Then, the transcripts were examined, and open coding was applied to individual quotes. Codes were then grouped into concepts and then into major themes. All transcripts were analyzed independently by two of the four investigators (MW, AvtS, AP, JL). To guarantee uniformity, MW was involved in the analysis of all group transcripts. Differences in opinion were discussed until consensus was reached. The final transcript yielded no new codes, indicating data saturation. Analyses were completed using Atlas.ti software.

## Findings

Of the 29 patient who were identified as discordant, all subsequently attended a focus group. The demographic characteristics of participants are summarized in Table 2.

**Table 2:** Demographic data for focus group participants

	Group 1 (*n* = 7)	Group 2 (*n* = 8)	Group 3 (*n* = 8)	Group 4 (*n* = 6)	Overall 1–4 (*n* = 29)
Female (no.)	5	5	7	6	23
Duration RA, years mean (IQR)	8	15.4	16.7	9	12.3 (4–10)
Erosive disease (no.)	1	4	4	4	13
Medication					
DMARD use (no.)	7	7	7	5	26
Biological use (no.)	2	6	3	3	14
Age, years (mean, SD)	57.5	56.1	58.7	55.2	56.8 (8.9)
VAS fatigue 0–100 (mean, SD)					67 (12.8)
HAQ, 0–3 (mean, SD)					1.1 (0.6)
RADAI, 0–10 (mean, SD)					2.7 (1.8)

Patients confirmed that they experienced high levels of self-reported disease activity despite the low disease activity reported by the professionals. If asked directly what could explain the difference between their own experience of high disease activity and the low disease activity according to the doctor, participants could not come up with a clear explanation. However, a clearer picture emerged during discussions of issues brought up by the participants themselves. Seven themes were identified during the inductive analysis, in line with our thematic analysis. In the following section, we elaborate on these themes. The quotes are identified by a participant number (e.g., P1) and a group number (e.g., FG1).

### Theme 1 perceived stress

The participants in our study indicated that higher levels of cognitive stress were associated with pain and functional disability. For example, if they appraised a situation as taxing and stressful and they considered themselves incapable of dealing with it, this increased the likelihood of experiencing symptoms.

Stressful situations at work or in private situations were discussed as an inducer of increased pain and fatigue:

Stress is also a big wrongdoer. And I have experienced a lot of stress for several years; and you do notice this, that it has a negative effect on everything, particularly the pain. (P2,FG4)

Besides the direct impact of stress, patients struggled how to cope with the high self-reported disease activity when they were feeling stressed.

Do you not also think that stress, unexpected events can have an influence on this? In my case they do. Like, for instance, the physical examination for my work is a very bad time for me, I feel that in my joints and everything. Because it’s on your mind a lot more. What will be the consequences, how will my employer react to this, how will I cope at home, how will I cope financially, so…. (P5,FG2)

Although they were well aware of the positive impact of low stress levels, it was hard to implement stress-reducing techniques. Patients reported feeling much better at times when they were able to cope with the pressure or during periods with low external stressors.

Another factor affecting stress was highlighted by specific remarks about the context of health care. If patients felt that their symptoms were not understood by healthcare professionals (including doctors) and therefore referred to other health professionals, this was experienced as stressful for some patients.

Being sent from pillar to post was very stressful for me (P8 FG2)

### Theme 2 balancing activities and rest

Patients mentioned that activities and rest need to be properly balanced in order to cope with the disease.

You have to find the right balance, and no-one can tell you what the right balance is between physical strain and relaxation (P4, FG2),

To manage activities, they used strategies such as planning, adapting, and avoidance of regular activities. In order to cope with the pain and fatigue that they expected to experience when taking part in specific activities, patients often made sure that they rested or relaxed in advance. Taking sufficient rest and restricting oneself in activities were hard for many patients. It was notable that patients regarded the need to make adjustments in daily life as a loss. They saw adjustments as an obligation rather than as an investment required to engage in valued activities. The need to rest was viewed as loss of usable time rather than energy renewal.

Another way of coping with high levels of disease activity was to ignore the symptoms and carry on. Some patients accepted that this would result in “off days”.

And a day like today, I couldn’t plan this on time, so for me this is actually already too much. I just know: tomorrow I’ll be ill. (P3FG2)

A number of patients also simply accepted ongoing high levels of disease activity as they did not wish to—or could not—reduce the level of their activities.

To exceed your limits, because either you can’t do it any other way or you don’t want to do it any other way. And that causes more problems the days after. (P4,FG1)

The patients also talked about how prior experiences with activities influenced how they made choices regarding current activities. Some patients avoid activities because of these bad experiences:

It is an obstacle that you don’t do things just because you know they will cause you pain later. (P6,FG1)

This not only changed their behavior but also made them feel that they were not able to do what “normal” people are capable of.

### Theme 3 medication intake

Most patients felt that medication had a negative influence on their general well-being that was not picked up by the physician’s measure of disease activity. They considered the medication side effects to be a potential cause of fatigue.

Get tired because of all those medicines. (P1,FG1)

Because of these side effects, some patients considered weighing the side effects against the severity of their disease in their decision whether or not to continue their medication.

At some point I really felt like a walking chemical factory.

Can you imagine: every week you inject MTX, you take proton pump inhibitors, Naproxen, Arava, an anti-malaria drug, and I was injected with boosts of prednisone. If you still manage to feel OK after that… terrible. (P2,FG4)

Other patients mentioned that it was hard to disentangle the symptoms caused by the disease from those caused by medication use.

### Theme 4 social stress

Social stress is defined as stress arising from a lack of social support or inappropriate social support.

Patients talked about the lack of social support and affiliation with family and friends. They felt that these people had limited understanding of their illness. Patients discussed feeling down related to the social response as a result of the invisibility of symptoms.

I can’t shake hands, I usually explain why. The common reaction of people is: It can’t be that bad, I don’t see anything (P2,FG3)

For some patients, a lack of understanding about the chronicity of the disease created negative thoughts, they always had to explain their condition resulting in more experienced stress.

I am being misunderstood by people around me; they keep asking: Have you still not recovered yet? I noticed that being misunderstood also causes me a lot of stress. (P4,FG2)

Some of them mentioned the same effect of feeling down if doctors ignored their pain and fatigue. Although most encounters only had a brief effect on patients, their recurrence made this a relevant topic for them.

Repeatedly having to ask for help was also difficult for some patients. It created a feeling of dependence on family or friends for everyday life. On the other hand, some patients found it difficult to deal with meddling by their family.

### Theme 5 relationship with professionals

The relationship with professionals and the professionals attitude was not regarded as having a direct negative effect on the self-reported disease activity, but it was suggested that good guidance, personal attention, listening, and taking time during consultations all alleviate self-reported disease activity.

If you leave the consultation room feeling badly, this will really affect you a lot. (P7,FG1)

Patients discussed the importance of being heard, and being listened to, were signs that professionals took them seriously.

Well, actually, it is not enough just to treat the illness; you have to treat the patient. And this patient is more than just that illness. (P2,FG2)

### Theme 6 comorbidity

Patients believed that comorbidity plays a part in the high disease burden, but it was difficult to interpret. Patients could not explain whether high self-reported disease activity was the result of RA or result of comorbidity. For example, if patients experienced pain in their joints they could not distinguish between osteoarthritis and arthritis .

And osteoarthritis is also very painful. And that also confuses me, when they ask: are your joints painful? Well, yes, they always hurt. (P6,FG 4)

A few patients discussed the causes of fatigue. Fibromyalgia or other diseases were mentioned as inducers of fatigue, but so was their RA. Female patients going through the menopause also talked about fatigue, disturbed sleeping and pain in the joint. But they could not say whether it was due to RA or menopause.

And menopause, that I have to open the windows, and then you’re still exhausted because it’s early in the morning and you’re still waking up. (P5, FG1)

### Theme 7 physical fitness

This theme was perceived as having a negative impact on well-being, both psychologically and physically. Patients mentioned that a lack of exercise led to more disease symptoms.

So when I sit down I get more overall complaints from my body; you become drowsy, you become tired. But when you move, your back doesn’t hurt as much, your hips don’t hurt as much, and so on (P5, FG 3)

Becoming unfit, that might worsen the illness. Well, worsen, maybe you just experience a symptom sooner (P6,FG1)

But the opposite was also reported: more pain and fatigue after exercise or other intense physical activities were mentioned as a reason for staying away from exercise. Although patients were aware of the benefits of exercise, their concerns about the pain and fatigue after exercising resulted in them avoiding all activities related to exercise.

If you exercise more, you get more energy. Well, it turned out to be so exhausting that I had to stop doing other things I liked. And it certainly didn’t give me more energy, so…it doesn’t help (P4,FG2).

## Discussion

### Main findings

We explored the discordance between the patients self-reported high level of disease activity, while at the same time, their doctors believed that their disease activity was low. Summarizing the data of the focus groups, we found seven themes that according to patients were relevant to high disease activity: perceived stress, balancing activities and rest, medication intake, social stress, relationship with professionals, comorbidity, and physical fitness.

### Comparison with existing literature

Considering the seven themes that emerged in our study, it seemed that the way in which patients coped with these themes may have played an important role in managing the impact of the disease on their daily life. Coping is the process by which people try to manage (e.g., reduce, minimize, master, or tolerate) the internal and external demands that are appraised as taxing or exceeding the resources of the person [22, 23]. Maladaptive coping may lead to loss of confidence, increased likelihood of perceiving a situation as stressful and loss of control [23], all of which hinder a person’s ability to adapt to living with a chronic illness [24, 25]. Results from RA studies suggest that problem-focused coping strategies are helpful in placing the disease into perspective, thereby allowing patients to better manage the disease burden [26]. Furthermore, studies have shown that active coping strategies appeared to be useful in RA patients and that these strategies improve psychological well-being [27].

Appraisal of the situation and employment of coping strategies differ considerably between patients. Some patients successfully adjusted to their disease while others did not, which is known from the literature [23, 28]. This was for example seen in physical activity, patients often tried to follow advice to do more exercise. However, some experienced more complaints hereafter. An explanation for this finding might be that initially RA comes with high levels of pain related to movement and exercise. These high levels of pain could lead to avoidance behavior, passive coping, based on fear of more pain [29] leading to a decrease in physical condition.

Also, the need for medication was viewed by some patients as a necessary evil with side effects implicating lower levels of health. From previous studies, we know that negative beliefs about medication can influence medication uptake, adherence, and the degree to which side effects are experienced [30, 31]. Patients with a more positive outlook on medication were more confident about the ability of DMARD treatment to control their RA [32]. It is therefore possible that negative thinking about medication has an impact on the experienced symptom reduction of known effective drugs (i.e., the nocebo effect).

At the same time, passive coping with pain and neglecting social support is known to have a negative impact on long-term disability and pain [33]. In this study, patients mentioned the importance of the support of professionals. This was in line with other studies where it was found that psychosocial factors influenced patient outcomes, but not disease activity [34, 35].

In the present study, we formulated the main question from the perspective of the professionals as we regarded low disease activity measured by DAS28 as treatment effectiveness. Some work has been done in this field by asking what patients perceived as disease remission. This resulted in themes such as absence or reduction of symptoms, ability to do valued activities and the ability to cope with the disease [36]. The patients’ and the doctors’ perspective on low disease activity thus appear to be based on different factors.

### Strengths and weaknesses

There are a number of limitations to this study. First, the results applied to patients with a median disease of 5 years. It is possible that patients with recently diagnosed RA would have identified different themes. Second, patients who were willing to participate were mainly Caucasian. It is possible that other ethnic groups would have discussed other themes. Third, we think that coping style may be seen as an important factor in the difference between physicians and patients assessment of the disease activity. Although coping style is often seen as a stable personality trait, coping style in chronic patients may be influenced by prolonged experience of stress that comes with rheumatoid symptoms. Therefore, coping style may have changed resulting in less optimal coping strategies. It is possible that our patients used less often problem-focused strategies, which hinder these patients to manage the burden in a more effective way [26]. This relationship could be bidirectional as prolonged experience of stress influences the presence of rheumatoid symptoms. This is not unlikely, because prolonged worry and anticipatory stress can influence somatic immunologic functioning [37].

Fourth, the focus groups consisted mostly of female patients (80 %). This could be the result of the fact that women are more likely to be discordant with the physicians [38]. However, male patients might have added different items which make that the results should be applied to female RA patients. Furthermore, depression was not measured in this study. Previous studies suggest a role for depression in patients with high levels of symptoms and estimated low disease activity. It is known that depression influences coping style, and thus depression could have influenced our results [33]. Although comorbidity was a theme of the discordance in disease activity, this study was not designed to specifically analyze this issue.

### Implications for clinical practice

Our results suggest that there are options for improving the quality of care for RA patients, which involve changing maladaptive coping styles by teaching them in problem solving techniques, transform negative thoughts (i.e., cognitive training), physical training, and the need for rest to renew energy. Doctors frequently see patients who had difficulties coping with their disease and who are discontented, despite the fact that, according to the same doctors, their disease activity is low. This discontentment may even lead to inappropriate drug treatment as patients may request to intervene in their symptoms, possibly initiating expensive drug instead of more appropriate non-pharmacologic management interventions. Specific interventions focused on one or more of the themes reported here may help to increase contentment, thereby improving not only the quality of life but also the satisfaction with the patient–doctor relationship for both parties.

When we asked patients why their view of their disease activity was different from that of their physician, seven themes emerged. The way in which patients coped with these themes in demanding situations may be the overarching theme.
